# Serotonin Metabolism and Serotonin Receptors Expression Are Altered in Colon Diverticulosis

**DOI:** 10.3390/medicina59111945

**Published:** 2023-11-03

**Authors:** Miłosz Jastrzębski, Piotr Nehring, Ilona Joniec-Maciejak, Adriana Wawer, Adam Przybyłkowski

**Affiliations:** 1 Department of Gastroenterology and Internal Medicine, Medical University of Warsaw, Banacha 1a, 02-097 Warsaw, Poland; milosz.jastrzebski@wum.edu.pl (M.J.); piotr.nehring@wum.edu.pl (P.N.); 2 Department of Experimental and Clinical Pharmacology, Centre for Preclinical Research and Technology, Medical University of Warsaw, 02-097 Warsaw, Poland; ijoniec@wum.edu.pl (I.J.-M.); adriana.wawer@wum.edu.pl (A.W.)

**Keywords:** biogenic amines, colonic diverticulosis, constipation, diarrhea, serotonin

## Abstract

*Background and Objectives:* Diverticulosis is frequently accompanied by altered bowel habits. The biogenic amines within colonic mucosa control bowel motility, and in particular, alterations in serotonin signaling may play a role in colon diverticulosis. The aim of the study was to assess the concentration of biogenic amines and serotonin receptor expression in the colonic mucosa in patients with diverticulosis and healthy controls. *Materials and Methods:* This prospective, comparative study included 59 individuals: 35 with sigmoid diverticulosis and 24 healthy controls. The study was held at the Department of Gastroenterology and Internal Medicine, Medical University of Warsaw, Poland. Mucosal samples were taken from the right and left colon during a colonoscopy in all patients. Concentrations of norepinephrine, 3-methoxy-4-hydroxyphenylglycol, dopamine, homovanillic acid, serotonin, and 5*-*hydroxyindoleacetic acid were measured with high-performance liquid chromatography. Expressions of human 5-hydroxytryptamine receptor 3A, 5-hydroxytryptamine receptor 4, 5-hydroxytryptamine receptor 7, solute carrier family 6 member 4 (SERT) for serotonin, as well as the neuroglia activation markers glial fibrillary acidic protein, S100 calcium-binding protein B, and proteolipid protein 1, were assessed with polymerase chain reaction. *Results:* The median age and sex distribution were comparable in both study groups (median 69 y vs. 52 y; *p* < 0.455 and males/females in cases 11/17 vs. 18/19 in controls; *p* < 0.309). In diverticulosis patients, there was a higher concentration of serotonin in the left affected colon compared to the right healthy part of the colon (median 8239 pg/mg vs. 6326 pg/mL; *p* < 0.01). The *SERT* expression was lower in the affected left segment compared to the right colon (median 0.88 vs. 1.36; *p* < 0.01). There was a higher colonic mucosa concentration of serotonin (median 8239 pg/mg vs. 6000 pg/mL; *p* < 0.02) and 5*-*hydroxyindoleacetic acid/serotonin ratio (median 0.27 vs. 0.47; *p* < 0.01) in diverticulosis patients compared to controls in the left side of the colon. *Conclusions:* The concentration of serotonin in the mucosa of the colon segment affected by diverticula is higher than in the healthy segment in the same individuals and higher than in healthy controls. These results underline serotonin signaling in colon diverticulosis pathophysiology.

## 1. Introduction

The prevalence of diverticulosis increases with age, reaching up to 60% at the age of 60 in Western and industrialized nations [1]. Diverticulosis shows both geographic and ethnic variability. In Western and industrialized nations, approximately 95% of patients with diverticulosis have diverticula in the sigmoid colon [2]. In Asia, the prevalence of diverticulosis is lower, reaching up to 25%, and diverticula localization is predominantly right-sided [3]. Diverticula localization may also vary by race. In the United States, both in Black and White populations, the most frequent localization of diverticula is the sigmoid colon, whereas diverticula distribution in the ascending colon and hepatic flexure is more frequent in Black compared to White patients [4]. The Asian population is characterized by a separate set of risk factors for colonic diverticula formation compared to Western nations. In the Japanese population, associations between colorectal diverticulosis and age, heavy alcohol consumption, cigarette smoking, and atherosclerosis have been documented [5]. Meanwhile, lifestyle (obesity, lack of exercise, low-fiber diet), abnormal colonic motility and high colonic intraluminal pressure, local inflammation, neuromuscular changes, and genetic factors are accepted risk factors for colonic diverticulosis in Western nations [6,7].

Colon motility is controlled by mediators released by enteric motor neurons, either through peristalsis stimulants (e.g., acetylcholine, substance P) or inhibitors (e.g., vasoactive intestinal peptide, nitric oxide). Signals both from and towards the central nervous system and spinal cord are transmitted to the gut not only by adrenergic (sympathetic or parasympathetic) routes, but also by dopaminergic or serotoninergic pathways. In the wall of the gut, neurons are concentrated mainly in the submucosal and myenteric plexuses.

Serotonin, or 5-hydroxytryptamine (5-HT), is an important monoamine in intestinal homeostasis, acting as a neurotransmitter and signaling molecule. 5-HT is derived from tryptophan, and in the intestines, 90% of it is produced by enterochromaffin (EC) cells, and 10% is produced in the myenteric plexus. The release of 5-HT from EC cells is regulated by multiple signals from the intestines, e.g., neuromodulators, toxins, pH, mechanical factors, and contraction. In EC cells, a vesicular monoamine transporter 1 (VMAT-1) sequesters 5-HT into granules that are transferred via the cell membrane and released with the help of the serotonin reuptake receptor (SERT) into the lumen of the intestine. Alterations in the serotoninergic system have been described in patients with diverticulosis, including increased mucosal expression of 5-HTR4s [8] and increased number of serotonin-positive cells [9,10], while diminished expression of SERT has been observed in diverticulitis patients [11].

Like the central nervous system, the enteric nervous system consists not only of neurons but also of glial cells. There is a growing amount of evidence that enteric glial cells (EGCs) play a pivotal role in modulating inflammation within enteral mucosa. Moreover, EGC activation, alteration, and destruction may be a response to intestinal permeability [12].

The sigmoid colon is the most susceptible segment of the colon to developing diverticula because of its small luminal diameter [13,14]. The ascending and proximal two-thirds of the transverse colon are derived from the midgut, while the distal transverse colon, descending colon, and rectum are derived from the hindgut. The diameter of the colon is around 8 cm at the level of the cecum and diminishes to 2.5–3 cm at the level of the distal sigmoid colon. Also, the blood supply of the right and left colon and rectum is executed by distinct arteries and veins. Finally, the small intestine and the large part of the colon are dependent on the ENS for their reflex control, while the distal part of the colon and rectum are dependent on the CNS. In the proximal colon, segmental constrictions of the circular muscle layer dominate, resulting in haustra formation, allowing proper stool passage. The sigmoid plays the role of gatekeeper for stool, and for that reason, the rectum is usually free of dwelling stool [15].

The study was triggered by the question of whether diverticulosis is accompanied by alterations in bioamine signaling. As the right and left part of the colon differ in embryonic origin, lumen diameter, innervation, and blood supply, the left and right colons were assessed separately to verify if there are intra- and interindividual differences in bioamines metabolism and serotonin receptor expression. The secondary aim of the study was to verify whether structural changes in the colon in patients with diverticulosis are accompanied by neuroglia activation in the human colonic mucosa in patients with colonic diverticulosis and healthy controls.

## 2. Materials and Methods

### 2.1. Patients

The study involved outpatients who had a colonoscopy for common colorectal symptoms in The Department of Gastroenterology and Internal Medicine, Medical University of Warsaw, Poland. This study was a prospective and comparative study; all patients who were examined with a colonoscopy for common colorectal symptoms were invited for participation in the study. The ROME IV questionnaire was completed by all study participants before colonoscopy to identify patients with functional disorders. In all patients, a colon mucosal biopsy was taken from the right and left colon, according to the study protocol. According to the results of the colonoscopy, patients were divided into two groups: colonic diverticulosis and healthy controls.

The inclusion criteria for the colonic diverticulosis group were left-sided diverticula diagnosed in colonoscopy. The exclusion criteria for studied groups included a history of intestinal resections, GI bleeding, cancer, pelvic or abdomen radiotherapy history or presence of diverticulitis, symptomatic diverticular disease, inflammatory bowel disease, irritable bowel syndrome, and history or current therapy with SSRI, SNRI, or pizotifen. The healthy controls comprised patients who had a screening colonoscopy negative for organic colon pathology in whom colonic diverticula were excluded in the colonoscopy.

All patients signed the informed consent for the study. The study was approved by the Ethics Committee of the Medical University of Warsaw, Warsaw, Poland.

### 2.2. Colonoscopy and Tissue Sampling

A colonoscopy was performed with the Evis Exera III CF-HQ190L Videocolonoscope (Olympus, Tokyo, Japan) under intravenous sedation. Mucosal samples of the right and left side of the colon were taken using biopsy forceps (Olympus). Tissue was immediately put into the Eppendorf^®^ Tube (Eppendorf AG, Hamburg, Germany) and stored at minus 80 degrees Celsius until analysis.

### 2.3. Neurotransmitter Concentration in Colonic Mucosa

The samples of intestine mucosa were weighed and homogenized using ultrasonic cell disrupter (Sonoplus HD 2070, Bandelin, Germany) in the mixture (1000 μL) containing 0.05 mM ascorbic acid and ice-cold 0.1 N perchloric acid (HClO_4_) and then centrifuged to precipitate proteins (Heraeus Labofuge 400 R, Heraeus Instruments, Hanau, Germany; centrifugation conditions: speed of 13,000× *g*, time of 15 min, temperature of 4 °C). Syringe membrane filters with pore size 0.2 μm (Puradisc; Whatman, UK) were used to filtrate the mixture. The supernatant was collected and used to perform biochemical analyses.

To determine the concentration of 3-methoxy-4-hydroxyphenylglycol (MHPG), norepinephrine (NA), dopamine (DA), homovanilic acid (HVA), 5-hydroxytryptamine (5-HT, serotonin), and 5-hydroxyindoleacetic acid (5-HIAA), a 20 μL aliquot of supernatant was injected into the high-performance liquid chromatography with electrochemical detection (HPLC-ED) system. The HPLC system consisted of a delivery pump (Mini-Star K-500; Knauer, Berlin Germany), an autosampler automatic injector (LaChrom L-7250; Merck-Hitachi, Darmstadt, Germany/Tokyo, Japan), an electrochemical detector (L-3500A; Merck-Recipe, Darmstadt/Munich, Germany) set at a potential of +0.8 V vs. a Ag/AgCl reference electrode. The mobile phase comprised 32 mM sodium phosphate buffer (Sigma-Aldrich, St. Louis, MO, USA), 34 mM citric acid buffer (Sigma-Aldrich), 1 mM octane sulfonic acid buffer (Sigma-Aldrich), 54 μM ethylenediaminetetraacetic acid (EDTA) buffer (Sigma-Aldrich) in ultrapure water (18 MΩ∙cm) containing 12% methanol solution (Merck, Germany). Separation of monoamines was achieved using EC 250/4 Nucleosil 100-5 C18AB (250 mm length × 4 mm internal diameter, 5 µm particle size, 100 Å) HPLC analytical column (Macherey-Nagel, Germany) and mobile phase flow rate maintained at 0.8 mL/min. Chromatograms were recorded and integrated by use of the computerized data acquisition Clarity software (version 5.0; DataApex, Prague, Czech Republic). Samples were quantified by comparison with standard solutions (external calibration). All used standards were purchased from Sigma-Aldrich. Assessment of HVA/DA and 5-HIAA/5-HT ratios had been previously introduced as methods to evaluate dopamine and serotonin metabolism [16].

### 2.4. Serotonin Receptor Expression

The mucosal mRNA expression of 5-hydroxytryptamine receptor 3A (*5-HTR3s*), 5-hydroxytryptamine receptor 4 (*5-HTR4*), 5-hydroxytryptamine receptor 7 (*5-HTR7*), solute carrier family 6 member 4 (*SLC6A4*, *SERT*), and glyceraldehyde-3-phosphate dehydrogenase (*GAPDH*) was measured using real-time PCR. Total RNA isolation was performed using TRI Reagent (Sigma-Aldrich) following the manufacturer’s protocol. The concentration of RNA was measured spectrophotometrically at a wavelength of 260 nm (BioPhotometer, Eppendorf AG, Hamburg, Germany). The single-strand complementary DNA (cDNA) was synthesized from total RNA using a PrimeScript RT Reagent (Perfect Real Time; Takara Bio, Otsu, Japan) in a SensoQuest Labcycler (SensoQuest GmbH, Göttingen, Germany). The incubation conditions for reverse transcription consisted of 15 min at 37 °C followed by 5 s at 85 °C. Following the reverse transcription reaction, the cDNA products were stored at −20 °C until further use. Real-time PCR was performed on a RotorGene Q 5plex HRM System (Qiagen Benelux BV, Venlo, The Netherlands). The cDNA was amplified with gene-specific primers designed using the publicly available National Centre for Biotechnology Information (NCBI) Primer-BLAST software tool https://www.ncbi.nlm.nih.gov/tools/primer-blast/. Target PCR primer sequences used in the study are shown in Supplementary Table S1. For all performed analyses, the reference gene was GAPDH. Based on previously published studies, the expression of serotonin receptor 3 (*5-HT3s*R) in the colon was evaluated by the expression of the *5-HT3s*A subunit. The expression of serotonin receptor 4 (5-HT4R) was evaluated by the expression of the “a” splice variant of this protein (*5-HT4a*). It was confirmed that this variant of the receptor was the most widely represented throughout the colon [17]. We amplified the housekeeping GAPDH gene in each sample to eliminate sample-to-sample differences during RNA extraction and conversion to cDNA. Each PCR mixture contained 1 μL of cDNA along with forward and reverse primer (1.25 μL of each primer), 10 μL of FastStart Essential DNA Green Master (Roche Molecular Systems, Alameda, CA, USA), and 6.5 μL of RNaze-free water (Thermo Scientific, Vilnius Lithuania) in a total reaction volume of 20 μL. The amplification protocol was as follows: initial denaturation at 95 °C for 10 min; 40 cycles at 95 °C for 15 s, 58 °C for 15 s, and 72 °C for 15 s. Melting curve analysis was applied to all reactions to ensure the consistency and specificity of the amplified product. All the amplifications were carried out in duplicate. The Pfaffl method was used to calculate the relative expression of the genes [18]. The following parameters were compared between groups: expression of a gene in the right and left colon, mean expression of the gene on both sides of the colon (arithmetic average of results from left- and right-sided colon), and mean difference in gene expression between both sides of the colon.

### 2.5. Neuroglia Activation Expression

The mucosal mRNA expression of the glial fibrillary acidic protein (*GFAP*), S100 calcium-binding protein B (*S100B*), and proteolipid protein 1 (*PLP1*) was assessed with the methodology described in Section 2.4.

### 2.6. Statistical Analysis

The data were assessed for normal distribution. For parametric and non-parametric variables, Student’s t-test and Mann–Whitney U tests were used, respectively. A regression model was used to assess differences in gene expression between samples and the coefficient of determination. Missing data were removed in pairs. The obtained results were considered significant if α < 0.05. All results were calculated using Statistica 13.3 software (StatSoft, Inc., Toulouse, KY, USA).

## 3. Results

### 3.1. Studied Population Characteristics

The study consisted of 59 consecutive patients who met the inclusion criteria and agreed to the study protocol: colonic diverticulosis (N = 35) and healthy controls (N = 24). All patients with diverticulosis were endoscopically assessed as stage 1 on the DICA classification (less than or equal to 3 points in the DICA score) and colonic diverticula were limited to the left side of the colon.

There were no significant differences between groups in terms of age, weight, height, and sex structure. The characteristics of the studied groups are presented in Table 1.

### 3.2. Neurotransmitters

In healthy controls, there were no differences in NA, MHPG, DA, 5-HT, HVA, and 5-HIAA concentration between the right and left colonic mucosa (Table 2a).

In the group of patients with diverticulosis, there was a higher concentration of 5-HT in the left colon compared to the right colon mucosa (median 8239 pg/mg vs. 6326 pg/mL; *p* < 0.01). There were no differences in colonic mucosa concentration of NA, MHPG, DA, HVA, and 5-HIAA between the right and left colon in diverticulosis patients (Table 2a).

On the right side of the colon, there were no differences in NA, MHPG, DA, 5-HT, HVA, and 5-HIAA concentration between diverticulosis cases and healthy controls (Table 2a).

On the left side of the colon, there was a difference in colonic mucosa concentration of 5-HT (median 8239 pg/mg vs. 6000 pg/mL; *p* < 0.02) and in the 5-HIAA/5-HT ratio (median 0.27 vs. 0.47; *p* < 0.01) between patients with diverticulosis and healthy controls (Table 2b).

### 3.3. Serotonin Receptors and Serotonin Transporter Expression

In healthy controls, there was no difference in *5-HT3s*, *5-HT4a*, 5*-HT7b*, and *SERT* expression between the right and left sides of the colon (Table 3).

In diverticulosis patients, there was a lower mucosal expression of *SERT* in the left colon than in the right (median 0.88 vs. 1.36; *p* < 0.01). Expression of 5*-HT3s*, *5-HT4a*, and *5-HT7b* receptors was comparable in the right and left part of the colon in diverticulosis patients (Table 3a).

In the mucosa of the right side of the colon, there was a difference in the expression of *SERT* (median 1.36 vs. 1.09; *p* < 0.02) between diverticulosis patients and healthy controls. There were no differences in *5-HT3s*, *5-HT4a*, and *5-HT7b* expression (Table 3b).

In the mucosa of the left side of the colon, there were no differences in *5-HT3s*, *5-HT4a*, *5-HT7b*, and *SERT* (Table 3b).

### 3.4. Colonic Neuroglia Activation

There were no differences in *GFAP*, *S100B*, and *PLP1* expression between diverticulosis patients and healthy controls in mucosa of the right and left side of the colon (Table 3a,b).

## 4. Discussion

We proved a higher concentration of 5-HT in the colon segment affected by diverticulosis. Our results are in agreement with the observations of Banerjee et al. [10], as they noted an increased number of 5-HT-positive cells in patients with complicated diverticular disease. In Banerjee et al., sixteen specimens of resected sigmoid with diverticulosis were compared with colon samples from the same patients not affected by diverticula. The study showed an increased number of serotonin-positive cells in specimens of sigmoid with diverticula compared to normal segments, indicating the possible role of serotonin in diverticular disease. The further study by Jeyarajah et al. did not evidence a difference in the number of 5-HT-positive cells in colonic mucosa between healthy controls and patients with diverticular disease [9].

Costedio et al. compared sigmoid colon biopsies in patients with diverticulitis, asymptomatic diverticulosis, and healthy controls [11]. That study consisted of the measurement of 5-HT content in colonic mucosa and mRNA expression of various genes. Costedio et al. observed reduced SERT expression in patients with a history of diverticulitis but not in patients with asymptomatic diverticulosis; the authors did not observe significant differences in 5-HT content and release [11]. On the contrary, in our study, we showed decreased SERT expression in colon segments affected by diverticulosis and increased 5-HT content. The reduced SERT expression in colonic mucosa accompanied by low values of 5-HIAA/5-HT ratio in diverticulosis patients suggests reduced reuptake and metabolism of 5-HT in affected colon segments and may explain elevated 5-HT mucosal concentration. Patients suffering from irritable bowel syndrome, which is a non-inflammatory condition, present diminished SERT expression within the colon; however, SERT downregulation was also reported in patients with inflammatory bowel diseases [19,20]. Taken together, this implicates SERT downregulation as a common feature in diseases of the colon.

Böttner et al. showed a difference in *5-*HT4 expression between affected and unaffected segments of the colon; however, they assessed resected fragments from patients with complicated diverticular disease and the study comprised only sixteen participants [8]. In contrast to the Böttner et al. results, in our study, we did not observe differences in *5-HT3*, *5-HT4*, and *5-HT7* expression between diverticulosis patients and healthy controls.

In recent years, chronic low-grade inflammation of the intestine was recognized as a risk factor for diverticulosis. Intestinal inflammation often increases the number of EC cells, which may result in higher concentrations of released 5-HT. The increased 5-HT concentration stimulates 5-hydroxytryptamine receptors (5-HTR) in immune and epithelial cells, resulting in decreased interleukin (IL)-4, IL-13, TNF-α (monocytes), and histamine, increased IL-1 beta, IL-6, IL-8, and IL-12p40, and TNF-α (macrophages), altered proliferation of B cells, increased dendritic cell migration, recruitment of eosinophils and neutrophils, epithelial adhesion, macrophage phagocytosis, and increased colonic transit. In a study of resected sigmoid segments in patients with a history of diverticulitis, the increased expression of *S100β* was observed in the submucosal and myenteric plexus. The *S100β* expression correlated with lymphocyte infiltration within mucosa [21]. Enteric glial activation may modulate neurotransmitter metabolism [12]. However, in our study, we did not observe differences in mucosal expression of neuroglia markers *S100β*, *GFAP*, and *PLP-1*. Moreover, mucosal *5-HT7* expression in patients with diverticulosis was comparable to healthy controls. Activation of *5-HT7* on dendric cells within the gut was previously suggested as a modulator of colitis [17,22]. Taken together, we may conclude that observed 5-HT and *SERT* changes in the sigmoid colon in patients with diverticulosis did not result from neuroinflammation.

In our study, we confirmed that dopamine content in colonic mucosa is extremely low compared to serotonin, even though DA has a proven influence on GI motility [23,24,25]. DA is synthesized, stored, and released vastly by neurons within myenteric and submucosal plexuses of the gut, which are usually beyond the reach of endoscopic biopsy forceps, which may be a possible explanation for the low concentration of DA we have observed. In the ascending colon of patients with Parkinson’s disease, the concentration of DA was found to be decreased, resulting in the impairment of GI function due to the loss of dopaminergic neurons in the CNS [26]. In our study, DA concentration was comparable within the studied groups.

The authors recognize a few limitations of the presented study. Firstly, due to the limited number of participants, the presented results are preliminary and require confirmation. Secondly, participants involved in the study had only the left colon affected with diverticulosis; therefore, studies also involving patients with extended diverticulosis should be performed. Thirdly, the presented study is limited to patients with diverticulosis, so studies enrolling patients with symptomatic diverticular disease and diverticulitis are reasonable.

In summary, our results show increased serotonin concentration and lower *SERT* expression in the colon segment affected by diverticulosis. Changes were not accompanied by mucosal neuroglial activation nor alternations of serotonin receptor expression. We hypothesize that serotonin signaling may play a role in colon diverticulosis pathophysiology; however, we are not able to conclude whether increased serotonin concentration in colon mucosa is primary or secondary to changes in sigmoid colon wall structure. The real nature of serotonin in the colonic segment affected by diverticulosis requires further studies.

## Figures and Tables

**Table 1:** Characteristics of the studied groups.

	Healthy Controls N = 28	Diverticulosis N = 37	*p*-Value
Age, y, median (IQR)	52 (44.5–66.5)	69 (63–75)	<0.455
Sex, male/female (%/%)	11/17 (39.3%/60.7%)	18/19 (48.7/51.3%)	<0.309
Height, cm, median (IQR)	168.75 (163.5–173.5)	164.5 (160–173)	<0.449
Weight, kg, median (IQR)	72 (60.5–82.5)	77.5 (65–84)	<0.788

IQR—interquartile range.

**Table 2:** (**a**) Comparison of bioamine concentration between the right and left colon in studied groups. (**b**) Comparison of bioamine concentration between groups on the right and left side of the colon.

(**a**)						
	**Healthy Controls**		***p*** **-Value**	**Diverticulosis**		***p*** **-Value**
	**Right** **Colon**	**Left** **Colon**		**Right** **Colon**	**Left** **Colon**	
NA, median pg/mg (IQR)	180	140	0.114	143	136	<0.39
	(133–209)	(93–192)		(103–224)	(98–199)	
MHPG, median pg/mg (IQR)	4.9	3.8	1.000	4.6	3.5	<0.411
	(1.8–11.2)	(1.8–13.3)		(1.9–15.6)	(0.9–9.6)	
DA, median pg/mg (IQR)	5.6	6.9	0.676	7.5	7.4	<0.565
	(2.4–11.2)	(2.9–10.6)		(2.8–15.5)	(3.8–12.2)	
HVA, median pg/mg (IQR)	2.8	3.4	0.965	2.7	3.1	<1.00
	(0.9–7.5)	(0.9–7.7)		(0.9–8.9)	(1.4–7.8)	
5-HT, median pg/mg (IQR)	6178	6000	0.982	6326	8239	<0.008
	(4679–7834)	(3774–8208)		(4278–8898)	(5727–12,051)	
5-HIAA, median pg/mg (IQR)	2849	2887	0.455	2709	3067	<0.881
	(1760.4–4071.6)	(1544–5725)		(1273–3858)	(1500–4583)	
HVA/DA	0.67	0.55	0.947	0.56	0.34	<0.511
	(012–1.20)	(0.12–1.81)		(0.21–1.30)	(0.13–1.04)	
5-HIAA/5-HT	0.53	0.47	0.524	0.45	0.27	<0.182
	(0.41–0.63)	(0.28–1.04)		(0.19–0.71)	(0.17–0.50)	
(**b**)						
	The right side of the colon		*p*-value	The left side of the colon		*p*-value
	Controls	Diverticulosis		Controls	Diverticulosis	
NA, median pg/mg (IQR)	180	143	0.386	140	136	<0.893
	(133–209)	(103–224)		(93–192)	(98–199)	
MHPG, median pg/mg (IQR)	4.9	4.6	0.943	3.8	3.5	<0.422
	(1.8–11.2)	(1.9–15.6)		(1.8–13.3)	(0.9–9.6)	
DA, median pg/mg (IQR)	5.6	7.5	0.509	6.9	7.4	<0.290
	(2.4–11.2)	(2.8–15.5)		(2.9–10.6)	(3.8–12.2)	
HVA, median pg/mg (IQR)	2.8	2.7	0.905	3.4	3.1	<0.893
	(0.9–7.5)	(0.9–8.9)		(0.9–7.7)	(1.4–7.8)	
5-HT, median pg/mg (IQR)	6178	6326	0.842	6000	8239	<0.020
	(4679–7834)	(4278–8898)		(3774–8208)	(5727–12,051)	
5-HIAA, median pg/mg (IQR)	2849	2709	0.893	2887	3067	<0.685
	(1760.4–4071.6)	(1273–3858)		(1544–5725)	(1500–4583)	
HVA/DA	0.67	0.56	0.905	0.55	0.34	<0.405
	(012–1.20)	(0.21–1.30)		(0.12–1.81)	(0.13–1.04)	
5-HIAA/5-HT	0.53	0.45	0.674	0.47	0.27	<0.020
	(0.41–0.63)	(0.19–0.71)		(0.28–1.04)	(0.17–0.50)	

**Table 3:** (**a**) Analyzed gene expression in the right and left colon for studied groups. (**b**) Comparison of analyzed gene expression between groups in the right and left half of the colon, respectively.

(**a**)				
**Healthy Controls**				
**Gene**	Right Colon *	Left Colon *	**R2**	***p*** **-Value**
*5-HT3s*	1.07 (0.53–1.39)	0.96 (0.35–1.57)	0.001	0.974
*5-HT4a*	0.91 (0.62–1.31)	1.03 (0.55–1.74)	0.001	0.872
*5-HT7b*	1.00 (0.70–1.39)	1.21 (0.75–1.63)	0.001	0.874
*SERT*	0.94 (0.71–1.44)	0.94 (0.65–1.60)	0.001	0.85
*GFAP*	0.77 (0.70–1.42)	0.77 (0.71–1.37)	0.001	0.805
*S100B*	0.85 (0.69–1.53)	0.95 (0.69–1.22)	0.001	0.529
*PLP1*	0.89 (0.61–1.17)	0.83 (0.67–1.68)	0.03	0.223
**Diverticulosis**				
**Gene**	**Right colon**	**Left colon**	**R2**	***p*** **-value**
*5-HT3s*	0.85 (0.48–1.90)	0.65 (0.41–2.15)	0.001	0.819
*5-HT4a*	1.08 (0.73–2.31)	1.01 (0.61–1.91)	0.01	0.4
*5-HT7b*	0.83 (0.63–1.50)	0.86 (0.63–2.38)	0.02	0.216
*SERT*	1.36 (0.75–2.08)	0.88 (0.64–1.43)	0.10	0.008
*GFAP*	0.99 (0.59–1.97)	0.80 (0.54–2.10)	0.001	0.984
*S100B*	1.01 (0.65–2.01)	0.82 (0.63–1.82)	0.001	0.867
*PLP1*	0.76 (0.60–1.22)	0.96 (0.61–1.85)	0.002	0.702
(**b**)				
**The right side of the colon**				
**Gene**	Healthy controls *	Diverticulosis *	**R2**	***p-*** **value**
*5-HT3s*	1.09 (0.58–1.39)	0.82 (0.48–1.82)	0.01	0.391
*5-HT4a*	0.96 (0.66–1.14)	1.08 (0.75–2.26)	0.03	0.202
*5-HT7b*	1.20 (0.75–1.38)	0.83 (0.63–1.50)	0.001	0.852
*SERT*	1.09 (0.80–1.55)	1.36 (0.75–2.08)	0.10	0.021
*GFAP*	0.96 (0.77–1.42)	0.92 (0.66–1.79)	0.02	0.285
*S100B*	1.02 (0.79–1.26)	0.95 (0.65–1.78)	0.02	0.236
*PLP1*	0.97 (0.74–1.32)	0.76 (0.60–1.22)	0.02	0.336
**The left side of the colon**				
**Gene**	**Healthy controls**	**Diverticulosis**	**R2**	***p-*** **value**
*5-HT3s*	1.03 (0.72–1.57)	0.73 (0.48–1.77)	0.01	0.564
*5-HT4a*	1.07 (0.77–1.66)	1.01 (0.65–1.80)	0.01	0.714
*5-HT7b*	1.28 (0.83–1.39)	0.89 (0.66–2.03)	0.02	0.221
*SERT*	0.99 (0.68–1.39)	0.86 (0.62–1.43)	0.01	0.701
*GFAP*	0.89 (0.76–1.33)	0.80 (0.54–2.14)	0.02	0.265
*S100B*	1.02 (0.83–1.32)	0.82 (0.63–2.21)	0.03	0.178
*PLP1*	0.93 (0.72–1.38)	0.95 (0.61–1.85)	0.01	0.773

* Gene expression relative to *GAPDH*, median (IQR); R2—coefficient of determination in linear regression.

## Data Availability

Dataset is available on reasonable request from the corresponding author.

## Supplementary Materials

The following supporting information can be downloaded at: https://www.mdpi.com/article/10.3390/medicina59111945/s1, Table S1: Target PCR primer sequences used in the study.
