# Supplementary material for: Serotonin Metabolism and Serotonin Receptors Expression Are Altered in Colon Diverticulosis
Source: Medicina (Kaunas). 2023 Nov 3;59(11):1945. doi: 10.3390/medicina59111945 (PMC10673248; doi:10.3390/medicina59111945)
Supplement: Supplementary file 1 [file medicina-59-01945-s001.zip › medicina-2681321-supplementary.pdf]

Table S1. Target PCR primer sequences used in the study.

Target	Accession No.	Real-time PCR Primer Sequences (5′-3′)	Predicted Product Size (bp)
HTR3A	BC004453.1	F: 5′-GCTGGGACATGAGGTTGGCAG-3′<br>R: 5′-GCTTTCCAAGCATAGGAGTGCC-3′	90
HTR4a	NM_001040169.2	F: 5′-ATGATGAGCGCTACCGAAGAC-3′<br>R: 5′-TGTGCAGAACGGTGTACCTTAGT-3′	105
HTR7b	NM_019860.4	F: 5′-TGTGCTGTAAGACAAAATGCTGAC-3′<br>R: 5′-ATGACTTCCTTCTGTTTCCACCTCT-3′	120
SLC6A4 (SERT)	NM_001045.6	F: 5′-ACCCTGACTTTTGGAGGGGC-3′<br>R: 5′-TTCGATCAGCGCGACAGTGA-3′	87
GADPH	NM_002046.7	F: 5′-TCCTGTTCGACAGTCAGCCG-3′<br>R: 5′-CCCCATGGTGTCTGAGCGAT-3′	70
S100B	NM_006272.3	F: 5′-ACAAGGAAGAGGATGTCTGAGC-3′<br>R: 5′-TCAGCTTGTGCTTGTCTCCCT-3′	97
PLP1	NM_001128834.3	F: 5′-GAAGCCCTCACTGGCACAGA-3′<br>R: 5′-CAGCAGGAGGGCCCCATAAA-3′	150
GFAP	NM_002055.5	F: 5′-GCAGGATGGAGAGGAGACGC-3′<br>R: 5′-GAGTGGAGGGGGCATTCGAG-3′	143
